# A system analysis of a suboptimal surgical experience

**DOI:** 10.1186/1754-9493-3-1

**Published:** 2009-01-06

**Authors:** Robert C Lee, David L Cooke, Michael Richards

**Affiliations:** 1 University of New Mexico, Department of Emergency Medicine, MSC10 5560, Albuquerque, NM 87131-0001, USA; 2 University of Calgary, Department of Community Health Sciences, Faculty of Medicine – Room G02, Heritage Research Medical Building, 3330 Hospital Drive NW, Calgary, AB T2N 4N1, Canada

## Abstract

**Background:**

System analyses of incidents that occur in the process of health care delivery are rare. A case study of a series of incidents that one of the authors experienced after routine urologic surgery is presented. We interpret the sequence of events as a case of cascading incidents that resulted in outcomes that were suboptimal, although fortunately not fatal.

**Methods:**

A system dynamics approach was employed to develop illustrative models (flow diagrams) of the dynamics of the patient's interaction with surgery and emergency departments. The flow diagrams were constructed based upon the experience of the patient, chart review, discussion with the involved physicians as well as several physician colleagues, comparison of our diagrams with those developed by the hospital of interest for internal planning purposes, and an iterative process with one of the co-authors who is a system dynamics expert. A dynamic hypothesis was developed using insights gained by building the flow diagrams.

**Results:**

The incidents originated in design flaws and many small innocuous system changes that have occurred incrementally over time, which by themselves may have no consequence but in conjunction with some system randomness can have serious consequences. In the patient's case, the incidents that occurred in preoperative assessment and surgery originated in communication and procedural failures. System delays, communication failures, and capacity issues contributed largely to the subsequent incidents. Some of these issues were controllable by the physicians and staff of the institution, whereas others were less controllable. To the system's credit, some of the more controllable issues were addressed, but systemic problems like overcrowding are unlikely to be addressed in the near future.

**Conclusion:**

This is first instance that we are aware of in the literature where a system dynamics approach has been used to analyze a patient safety experience. The qualitative system dynamics analysis was useful in understanding the system, and contributed to learning on the part of some components of the system. We suggest that further data collection and quantitative analysis would be highly informative for identification of system changes to improve quality and safety.

## Background

Patient safety and quality of care are crucial issues in health systems worldwide. Despite frequent calls for a systems approach to patient safety [1-3], systems analyses of incidents that occur in the process of health care delivery are rare. The retrospective nature of typical assessments of incidents necessitates use of simple methods such as root cause analysis [4], which, although informative, does not model system interactions and how a system changes over time. In contrast, system dynamics modeling [5] is a powerful and flexible means to represent complex systems and interactions. System dynamics has been applied to a limited degree in health care in general [6-11], and to a very limited degree in patient safety [12], but a search of the PubMed online database did not result in any published explorations of actual patient safety events.

The unfortunate experience of one of the authors provided a case study for which a qualitative system dynamics model could be built. This patient/author is a patient safety researcher who works within an academic hospital system, and thus was able to observe events with perhaps more fidelity than a typical patient; plus he had the additional observational advantage of a spouse who is also a researcher and who accompanied him throughout the process of care. We interpret the sequence of events as an illustrative case of cascading incidents that resulted in outcomes that were less than optimal, although fortunately not fatal. Incidents, in this paper, are defined as deviations from an optimal pathway of care. In this sense, incidents not only include errors or mistakes, but missed diagnoses or conditions, delays in treatment, etc. [13]. Such incidents can inform how systems perform in a suboptimal fashion.

The patient was subject to a routine outpatient procedure: removal of an epididymis due to the existence of large cysts (i.e. spermatoceles). The procedure was performed under general anesthesia by a senior urology resident, under observation by an attending urologist. No drains were placed. The patient was sent home with pain medication, antibiotics and a scrotal support packed with gauze. Table 1 is a sample of incidents that resulted in pain and discomfort, worry, and additional encounters with the health care system.

**Table 1:** Incidents that occurred during the process of care

**Step in process**	**Department**	**Incident**	**Outcome**
*Preoperative assessment*	Surgery/Urology	Failure to warn or notify patient to stop gabapentin*	Hemorrhage, purpura (after surgery)

*Surgery*	Surgery/Urology	Failure to stop bleeding	Hemorrhage, swelling, pain, worry
		
		Failure to install drains	Swelling, pain

*Post-operative process*	Emergency	Failure to be seen by physician	Delay, swelling, pain, worry
		
		Failure to call urology resident	Delay in being seen
		
		Failure to triage and to recognize ongoing bleeding	Near syncope
	
	Surgery/Urology	Lack of beds	Waiting without supervision (risky for many reasons)
	
	Emergency, Surgery/Urology	Inexperienced residents recommending overly conservative "watch and wait"	Continued swelling, purpura, pain, worry
		
		Attending not seeing patient	Expert assessment not provided

*Second preoperative assessment*	Surgery/Urology	Released without being seen	Expert assessment not provided

*Second surgery*	Anesthesia	Failure to warn or notify patient to stop gabapentin	Longer recovery

	Surgery/Urology	Inexpert IV insertion	Hemorrhage, purpura (in arm)

		Long wait for surgery	Swelling, pain, worry

*Note that gabapentin can increase vasodilation and purpura in 1% of patients. The health system of interest appropriately asks patients at multiple points in the process of care as to what medications they take; however, there was never a suggestion that the patient should stop taking this drug.

The patient experienced a large degree of scrotal swelling (eventually up to approximately 15 cm), and subcutaneous spread of blood down both legs to the knees and up to the mid-waist. As instructed and as typical in the health system of interest, the patient went to the emergency department (ED) and asked that the urology resident on call be summoned. The emergency department was crowded, and the hospital had no beds available at the time. The patient called the resident himself as the pain and swelling persisted, his heart rate increased, and he became faint. He was never seen by an emergency physician. The urology resident arrived later, then called a more senior resident; they took the conservative "watch and wait" approach. After an extremely uncomfortable night at home, the patient called the urology department the next morning and was seen by senior residents and an attending physician. He was re-admitted to surgery (after many hours of delay due to scheduling issues), whereupon a large clot was removed, a leaking artery tied off, general bleeding cauterized, and drains installed. Ultrasound indicated that blood flow to the testicle had not been compromised. Other than a large degree of internal scarring, the eventual outcome was acceptable.

An important point is to not view the incidents in Table 1 as a "laundry list" of complaints, but rather as symptoms of system problems. We can use the patient's experience along with some simple models to gain insights into the behaviour of the system. These insights suggest system improvements.

## Methods

In the field of system dynamics, a process of qualitative and quantitative modeling has been developed to inform difficult issues to the degree possible [5]. The system dynamics method is a good way to approach the dynamic complexity of health systems [14]. "Problem articulation" is the first step. In this case, a system exists in which it is possible to have a series of events that result in suboptimal care. It could be argued that the entire health system of the US can be characterized in this way, but our intent is not to model this entire system; rather, we will focus on a subsystem that led to the incidents of interest. Likewise, our focus is not on a cohort of patients or the entire medical history of the patient of interest; rather we will focus on a time block of several days over which the events of interest for the patient of interest took place. By focusing the problem thusly, we can explore it to a greater level of detail.

A "dynamic hypothesis" is "a working theory of how the problem arose" [5]. In this paper, we develop a dynamic hypothesis using patient flow diagrams. The flow diagrams were constructed based upon the experience of the patient (as recorded during and after the events), medical chart review, discussion with the physicians involved with his care as well as several physician colleagues, comparison of our flow diagrams with those developed by the hospital of interest for internal planning purposes, and an iterative process with one of the co-authors who is a system dynamics expert. Note that our intent is not to develop a fully functional quantitative simulation model, as this is not possible given current availability of data, but rather to structure the problem to clarify some issues that may have intuitive meaning for administrators and policy makers, and which may suggest some "fixes".

The patient flow diagrams are made up of three basic types of variables, using standard nomenclature as applied in the system dynamics field:

1. A "stock" of patients, shown by a variable in a rectangular box. Typically a stock variable describes the state of the patient at that point in the process.

2. A "flow" of patients from one stock to another, shown by a variable above a "flow valve" symbol. Flows across system boundaries are denoted by "cloud" symbols.

3. Auxiliary variables that influence the flows – typically these are capacities, resources, delay times, etc.

Arrows are used to denote a causal relationship between variables.

## Results

### The outpatient surgery system

**Figure 1:**
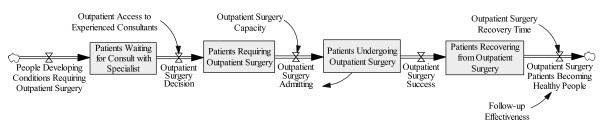
**The Outpatient Surgery System**.

The urology surgical system that the patient encountered is likely to be similar to many outpatient surgery systems for medical specialties. Ideally, everything goes well and the patient flow through the outpatient surgery system is as shown in Figure 1.

The flow of People Developing Conditions Requiring Outpatient Surgery is continually maintained by disease progression and events occurring randomly in the general population. People become "patients" when their condition reaches the point of requiring medical attention. When a family physician determines that the condition requires surgery, a referral is made and the patient goes onto a waiting list for outpatient surgery, here called Patients Waiting for Consult with Specialist. This stock is depleted by an Outpatient Surgery Decision, controlled by the access to consultant resources, here called Outpatient Access to Experienced Consultants. Non-surgical treatment decisions, not shown in the diagram, would also deplete this stock and move such patients outside of the system boundary. The flow of Outpatient Surgery Admitting is controlled primarily by Outpatient Surgery Capacity. Note that surgeries are routinely cancelled and thus delayed, largely due to scheduling/capacity issues. After successful operations, patients leave the stock of Patients Undergoing Outpatient Surgery, which frees up space for more patients to be admitted. Ideally, Patients Recovering from Outpatient Surgery are monitored by the surgeon or staff to ensure a successful recovery, but the natural healing process (denoted by the variable Outpatient Surgery Recovery Time) determines the rate of Outpatient Surgery Patients Becoming Healthy People. If the surgery is not successful or untoward outcomes result, the patient may be admitted to a hospital.

If the patient of interest had experienced the Outpatient Surgery System as described above, all would have been well and this paper would not have been written. However, the patient did not have an ideal experience, and as a result also experienced the ED system, which will be described next.

### The ED system

**Figure 2:**
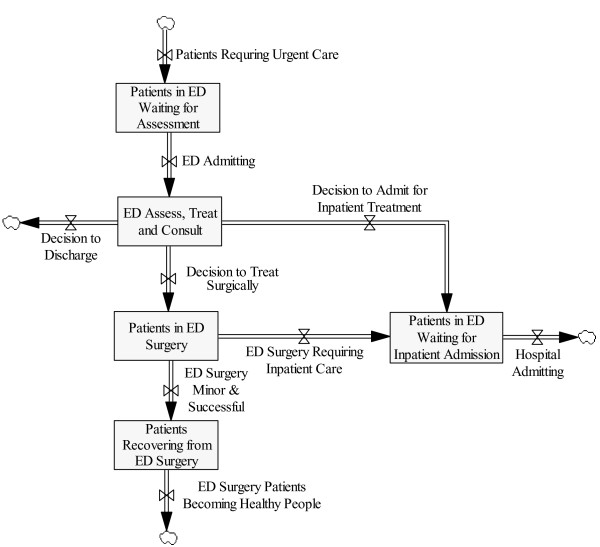
**The ED System**.

The patient flow paths in the ED system are shown in Figure 2. Briefly, "urgent patients" are defined here as both those at immediate risk of serious morbidity or death (i.e., emergent patients), as well as those with medical conditions that have the potential to escalate to emergencies. These patients in the health system of interest present to a paramedic, who registers the patient, and takes vital signs and chief complaint(s). If the patient requires resuscitation, they are "fast-tracked" to the resuscitation room. Remaining patients (after waiting) are triaged, and the subsequent process depends upon the triage score and their particular condition.

In a typical system, patients are admitted when a treatment space becomes available and the triage nurse determines that the patient is the next highest priority to be seen. The ED of interest is also used essentially as a referral center for consults with specialists (as in the case here), and is also a source of primary care for a large urban uninsured population, thus it is subject to capacity and flow issues.

### Interaction between the two systems

**Figure 3:**
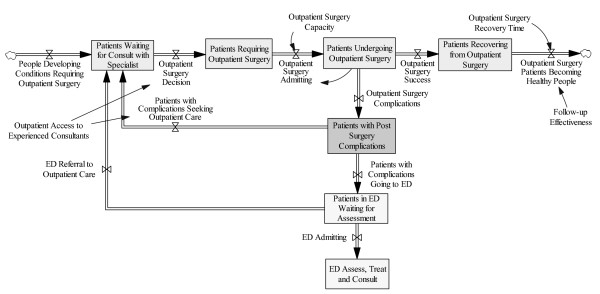
**Simplified View of ED System/Outpatient Surgery System Interactions**.

Some of the problems that the patient encountered arose because of the nature of the interaction, or in some cases, lack of interaction between the two systems. Figure 3 shows the system interactions, with the ED system shown only up to ED Assess, Treat and Consult.

The outpatient surgery system becomes "connected" to the ED system when there are post-surgery complications. In the case of post-surgical complications, the patient actually leaves the normal outpatient surgery system and does not enter a state of normal recovery. The Patients with Post Surgical Complications are in limbo between the two systems. The route of Patients with Complications Going to ED is dictated by the patient's or advocate's sense of urgency and the availability/capacity of the health system of interest. If the patient feels the complication does not require immediate attention, the route may be Patients with Complications Seeking Outpatient Care. In the present case, the patient had a sense of urgency and the ED system was the only option available. For Patients in ED Waiting for Assessment, control of the decision for ED Admitting versus ED Referral to Outpatient Care falls under the ED system. In this case, there was no other choice given to the patient (although in retrospect the patient could have perhaps worked around this by aggressive personal interaction with the urology resident on-call). The system in Figure 3 is somewhat idealized because it assumes a simple emergency/non-emergency decision controls the choice of flow path out of Triage. In reality, there are system constraints and system push-back that often prevent patients from accessing the ED system in an effective and timely fashion.

**Figure 4:**
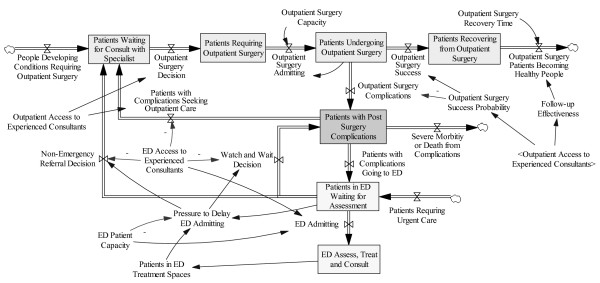
**A More Realistic View of System Interactions**.

In Figure 4, we add another recycle loop called Watch and Wait and add several of the important factors that were influencing system flows in this patient's case. We also add the important outflow called Severe Morbidity or Death from Complications. In this case, the most likely severe consequence would have been testicular necrosis, but it would have been possible for other severe outcomes or death to have occurred. For example, if the patient exacerbated the condition by taking aspirin or non-steroidal anti-inflammatory drugs for pain, was mentally or socially compromised, and became frustrated so that he left without being seen and did not follow up; the bleeding could have progressed to a point where the stitches burst and the patient bled to death.

Figure 4 also shows that patients in this system who are suffering from post-surgery complications are competing for ED resources with other Patients Requiring Urgent Care. Some (or many) of these patients may have more urgent conditions than the patient of interest, and, given the evidence that there was waiting room overcrowding when the patient arrived, there is pressure to delay admitting and adopt a "watch and wait" approach. This is captured in the system diagram (Figure 4) by the Pressure to Delay ED Admitting variable. The ED Admitting flow is zero unless Patients in ED Treatment Spaces are less than ED Patient Capacity. In other words, an existing patient has to leave before a new patient can be admitted. It is well known [6,7] that ED beds can be blocked by downstream bottlenecks. For example, Patients in ED Waiting for Inpatient Admission (Figure 3) can block ED beds until inpatient space becomes available. Inpatient beds can also be blocked by other means, such as sick elderly patients who cannot leave the hospital because there are no long term care spaces available.

Another factor influencing the likelihood of a "watch and wait" approach is the ED Access to Experienced Consultants. It is unreasonable to expect that an ED can provide immediate access to attending consultants of every kind. In a typical US teaching hospital, resident consultants are on-call and an attending physician consultant would only be called in to accept the case if the attending ED physician convinced the attending consultant that the patient required immediate specialized medical attention. In this patient's case, 1) the ED failed to call the resident, and thus the patient did so after a period of frustration, and 2) when he arrived, the on-call resident had no experience with the sort of morbidity that was exhibited, thus he called a more senior resident. This resident again was unsure as to appropriate action, and called an attending urologist. This urologist made the decision to "watch and wait" until the next day. There were no ED beds available, so rather than spend the night sitting in an uncomfortable chair the patient elected to go home at this point. In the diagram, the residents/attending thus made a Non-Emergency Referral Decision and the patient was routed back to the Outpatient Surgery System.

The bleeding was addressed by the second surgery, and drains were placed in the scrotum. Subsequent to the surgery, the patient had a large amount of bruising at the site of the intravenous drip that was placed (a result of inexpert placement), and a painful throat due to inexpert intubation. As a result of the large amount of swelling and fluid accumulation, the patient had to manage the drains for more than 2 weeks. Health economists use a measure called health-related quality of life (HRQOL) [15] to quantify not only benefits of treatment, but discomfort, pain, suffering, and other undesirable outcomes of medical care. The patient, if given one of these questionnaires at the time, would have rated his HRQOL as very low for approximately two months during and after the surgery. Fortunately, the only permanent negative outcome was internal scarring. If the patient had lost a testicle, developed infections, etc. post surgery; it could have been much worse.

### Dynamic hypothesis

Like many adverse events in complex systems, this one has no single root cause but rather arose from a multi-layered, time-related combination of system failures. Our hypothesis is that these failures originated in the design of the system, and in the many small innocuous system changes that have occurred incrementally over time, which by themselves may have no consequence but in conjunction with some system randomness (e.g., "bad luck") can have serious consequences. For example, the past decision to use the ED as a referral center for consultants outside of normal business hours, which may be efficient in some instances or may have been at one time, was a major source of delay in this case due to the patient "falling between the cracks" on a busy night. The numerous other changes that have likely occurred over time are beyond the scope of this paper, but would constitute an interesting study.

In the patient's case, the incidents that occurred in preoperative assessment and surgery (see Table 1) originated in communication and procedural failures. System delays, communication failures, and capacity issues contributed largely to the subsequent incidents. Some of these issues were controllable by the physicians and staff of the institution (e.g. "failure to stop bleeding", "failure to call urology resident"), whereas others were less controllable (e.g. "lack of beds", "long wait for surgery"). To the system's credit, some of the more controllable issues were addressed (see below), but systemic problems like overcrowding are unlikely to be addressed in the near future.

## Discussion

We have presented a qualitative system model of a series of incidents that were tracked by the patient of interest and his advocate, confirmed via chart review, and analyzed by the patient and colleagues. These incidents occurred despite the fact that the patient was familiar with the system and many of the staff; plus he had an informed and aggressive advocate in the form of his spouse who accompanied him throughout the process of care (the patient himself had difficulty thinking at times due to discomfort and the large amount of pain medication he was taking); i.e., "it can happen to anyone". The consequences for a less informed or capable patient (e.g., a non-English speaker, an alcoholic, etc.) may have been much more severe.

This experience provides an excellent case example of system failures and inefficiencies that fortunately resulted in transient negative outcomes (as opposed to serious permanent injury or death), and allowed the patient and his colleagues to document and analyze the case. Please note that this case is by no means unique or limited to the health system of interest; every patient population and hospital system in the US, and indeed worldwide, is subject to such system failures.

After recovery and return to work, the patient wrote a brief note to the Chairs of the Departments involved, describing the events (similar to Table 1) and suggesting how the state of the system, including communication between Departments, might be improved. The Chairs were appreciative of the note, and noted that they were taking the case seriously.

In general, hospital systems have not been designed using operations management and industrial engineering methods that have been applied in many other industries, with the primary goals of efficient production, quality and safety. Although many hospital systems recognize that these methods should be applied (as evidenced by numerous patient safety papers and reports in the literature (e.g., [2,6,8,10,11,14,16,17] and available courses and lectures on the subject; e.g. [18]), there are many barriers to implementation [19]. In many cases, recognition that hospitals are institutions that should have quality and safety as primary goals is sadly lacking.

In the system of interest here, three factors will likely impede major organizational changes in the near future. The first is the fact that, on a typical day, dozens of similar failures of the system likely occur; some less, and some much more serious. Some result in no action on the part of the patients, as they may be unaware that the negative consequences of their care were outside of the norm, or they may have come to expect that their encounters with a health system will be suboptimal. At the other extreme, some result in major lawsuits and/or large settlements. However, it is unlikely that many components of the health system will ever reach the state of "ultrasafety" that other industries have achieved [19], especially in a case such as this where multiple departments are involved and interact. The key is to learn from incidents and to improve the system to the degree possible [20,21]. Although there was limited learning as a result of this incident, at present the health system of interest does not have a formal incident learning (as opposed to simple reporting) system in place; this will be required for substantial improvement.

The second factor that will impede change is that the health system of interest, as related to the incidents described, has an entrenched policy in which the ED is used as a primary care center and a referral center in addition to its intended use for urgent and emergent care. This again is a difficult issue. Simply providing, for example, urgent care centers as clearinghouses would not solve the problem, as staffing these centers would be problematic given current chronic staff shortages nationwide. Although there was some risk in the clinical decision that the patient's condition was not an emergency (e.g., he could have gone the Severe Morbidity or Death from Complications route), the overall system requires checks and balances to prevent abuse. In this case, the ED was the only referral system that the patient could access outside of normal working day hours. However, if people perceived that ED Access to Experienced Consultants during normal working hours was greater than Outpatient Access to Experienced Consultants, then more people would go to ED to seek medical attention for non-urgent conditions. System designers should take considerations such as this into account. The patient could have, in retrospect, perhaps used personal contacts and workarounds to improve his personal care, but that would do nothing to improve the system.

The third factor is the patient went to a teaching hospital for his surgery. Teaching hospitals provide a valuable service; i.e., training of students and residents, but in this case the hierarchical system of assessment and treatment by junior and senior residents introduced delays and failures in the system that contributed to the problem. Again, the patient could have perhaps insisted upon seeing an attending physician at all times, but this would likely have been infeasible in terms of availability of those physicians. He could have gone to a private hospital, but in the city of interest the private hospitals seem to have similar wait time, delay, and quality issues as the teaching hospital. These issues would best be informed by careful operational assessment of the system, using tools such as quantitative system dynamics, but also discrete event simulation and optimization [7,22,23]. These sorts of models require data and/or expert opinion on a large number of variables, and it would often be necessary to supplement existing information collection systems in order to build meaningful models. Success is dependent upon availability of resources in a financially constrained system. Regardless, data collection and modeling are likely to be highly cost-effective both in terms of increases in efficiency and perhaps avoidance of lawsuits.

Some changes in the system were prompted by this patient's experience, due to both the willingness of the system to accept change as well as the initiative of the patient in bringing system deficiencies to the light of day. The ED has instituted a "patient arrival form", which includes documentation of any referrals that are necessary. This form is provided to both the charge nurse and to triage. The urology division presented the patient's case at its morbidity rounds, and the senior residents informed the patient that after this experience that they would always place drains after similar surgery. Provision of information similar to Table 1 to both departments helped clarify some of the system problems; this paper should bring further clarification to a wider audience.

## Conclusion

In conclusion, we have presented a qualitative system dynamics analysis of a series of incidents that affected one of the authors, and which involved interactions across departments. The system dynamics approach applied here, although qualitative, allows more extensive analysis of a complex system than many other methods currently applied in health care. Ideally, given further data collection and more cases to evaluate, a quantitative computational model could be built and employed to model the system in more detail and with greater generalisability. Computational system dynamics methods are able to evaluate policy changes and 'what-if' scenarios that can be highly informative for decision-makers. As application of system dynamics in patient safety is in its infancy, we are currently exploring development of a computational model and application to a wide variety of safety issues in a research setting. Once the methods have been 'tested' on a variety of scenarios, and the reliability of the model evaluated, we could then explore more routine use of the model in informing safety programs as well as more general operational aspects of the system.

We suggest that both qualitative and quantitative system dynamics approaches can and should be added to the 'toolbox' of methods that are being applied to patient safety problems in health systems worldwide. System dynamics provides a means to engage clinical staff and administrators in assessing and understanding complex safety issues and possible solutions, and thus potentially facilitates buy-in and action.

## Competing interests

The authors declare that they have no competing interests.

## Authors' contributions

RL conceived the study and participated in its design, collected information used in the study, and led writing of the manuscript. DL participated in design of the study, led construction of the models, and participated in writing of the manuscript. MR participated in design of the study, provided clinical expertise and review, and participated in writing of the manuscript.
